# pcPromoter-CNN: A CNN-Based Prediction and Classification of Promoters

**DOI:** 10.3390/genes11121529

**Published:** 2020-12-21

**Authors:** Muhammad Shujaat, Abdul Wahab, Hilal Tayara, Kil To Chong

**Affiliations:** 1 Department of Electronics and Information Engineering, Jeonbuk National University, Jeonju 54896, Korea or mshujaat.bulc@bahria.edu.pk (M.S.); heroaw2018@jbnu.ac.kr (A.W.); 2 Department of Computer Sciences, Bahria University, Lahore 54000, Pakistan; 3 School of International Engineering and Science, Jeonbuk National University, Jeonju 54896, Korea; 4 Advanced Electronics and Information Research Center, Jeonbuk National University, Jeonju 54896, Korea

**Keywords:** bioinformatics, computational biology, convolution neural network (CNN), promoters, non-promoters

## Abstract

A promoter is a small region within the DNA structure that has an important role in initiating transcription of a specific gene in the genome. Different types of promoters are recognized by their different functions. Due to the importance of promoter functions, computational tools for the prediction and classification of a promoter are highly desired. Promoters resemble each other; therefore, their precise classification is an important challenge. In this study, we propose a convolutional neural network (CNN)-based tool, the pcPromoter-CNN, for application in the prediction of promotors and their classification into subclasses σ70, σ54, σ38, σ32, σ28 and σ24. This CNN-based tool uses a one-hot encoding scheme for promoter classification. The tools architecture was trained and tested on a benchmark dataset. To evaluate its classification performance, we used four evaluation metrics. The model exhibited notable improvement over that of existing state-of-the-art tools.

## 1. Introduction

Promoters are short DNA sequences located near the beginning of the gene’s transcription site. Promoters play a significant role in initiating the process of transcription in genes. Throughout the gene transcription process, s (sigma) is an important factor of RNA holoenzyme that identifies the promoter sequence. Bacterial promoters are composed of purines at the transcription start site (TSS). At the TSS, the hexamer TATAAT is centered at −10, while TTGACA is centered at −35 [1]. The RNA polymerase of the bacterium *Escherichia coli* has many sigma factors, factors that are dependent on environmental factors and gene identity.

Different types of promoter sequences are identified by different sigma factors; therefore, the type of s factor decides the category of the bacterial promoter. Sigma factors are divided into six different types: σ70, σ54, σ38, σ32, σ28 and σ24 where each sigma factor has different functions. The σ28 factor is responsible for flagellar gene function during normal growth, whereas σ24 and s32 are responsible for the heat shock response and the exponential growth to the stationary phase in *E. coli*. The σ38 factor is associated with the stress response during transition [2] and σ54 is involved in the regulation of nitrogen metabolism [3]. The most important sigma factor and the one that is obligatory for transcription commencement in most genes is σ70. A recent study has shown that σ70 can also affect RNA polymerase activity during elongation [4].

The available biological methods for promoter classification are time-consuming and involve undertaking an expensive procedure. Usually, a promoter can deviate from position to position; therefore, it is challenging to effectively identify a promoter by applying biological techniques [5]. Regardless, the accurate identification of a promoter is essential in the formulation of every gene and transcription unit within the genome. To overcome the disadvantages of biological classification methods, computational techniques for predicting promoter function have been developed.

Over the last few years, various computational techniques for different research problems have exhibited great results [6,7,8,9,10]. Similarly, computational techniques have been developed to classify DNA sequences as either promoter or non-promoter regions, and some techniques are reported to identify the specific sigma class of a promoter. For example, ref. [11] introduced a sequence-based identifier that could predict the presence of a σ70 promoter. The proposed method, PseZNC, formulates the DNA sequence based on nucleotide composition. Besides, a variable-window Z-curve method to identify promoters was presented by [12]. The BacSVM+ software package, which is based on the LibSVM library, was reported to predict promoters from *Bacillus subtilis* [13]. Also, De Avila e Silva et al. [14] developed a method to predict the σ28 and σ54 promoters in *E. coli* that was based on the duplex stability feature of the neural network. A deep feature selection method proposed by [15] evaluates the non-linearity of a deep structure and selects a subset of the deep feature at the input level to predict promoters within a DNA sequence. Le et al. [16] presented a hybrid technique that combined deep learning and FastText N-grams to predict promoters and their strengths. Finally, Rahman et al. [17] introduced a technique based on a feature subspace-based ensemble classifier to predict σ70 promoter sequences.

To investigate the sequence features of prokaryotic and eukaryotic promoters in *E. coli*, a convolutional neural network (CNN)-based architecture was proposed in previous research [18]. In [19], Liu et al. introduced a model named iPromoter-2L, which included a two-layer prediction model in which the first layer predicted whether the DNA sequence is a promoter or non-promoter, and the second layer identified the promoter class from among sigma classes σ70, σ54, σ38, σ32, σ28 and σ24. The report by Zhang et al. [20] addressed the same research problem and proposed a model named MULTiPly that can improve the predictive performance of previous techniques. MULTiPly is a multilayer dual-task predictor that can distinguish between a promoter and non-promoter and can identify promoter class. MULTiPly first identifies the best combination of information features by using an F-score feature selection method; that step is followed by applying five binary classifiers to identify the promoter class. A model named iPromoter-BnCNN proposed by Amin et al. utilized four parallel one-dimensional convolutional filters applied to the monomer nucleotide sequence, the trimer nucleotide sequence, and the structural belonging dimers and trimers of the DNA sequence [21]. The dense layer combined all of the extracted features and performed the classification task. The proposed model was applied to *E. coli* to predict promoters and non-promoters and promoter sigma classes. It showed improved results compared to results from MULTiPly and iPromoter2L.

Recent computational methods for the identification and classification of sigma promoters have shown a marked improvement in sensitivity, specificity, accuracy and Matthews correlation coefficient (MCC), but there is still room for improvement. For example, the performance of the iPromoter-2L tool produces conflicting results for classification sensitivity and specificity. The MULTiPly method tried to overcome that problem, but limitations in the selection of basic features remain. The iPromoter-BnCNN performed step by step binary classification and showed impressive results when compared with those previously reported. Overall, that method achieved 88.2% accuracy, 88.3% sensitivity, 88.0% specificity and 0.763 MCC. Regardless, the main limitation of iPromoter-BnCNN is the extraction of local features and structural properties.

In this study, we present a pcPromoter-CNN model. The pcPromoter term stands for “prediction and classification of promoters”. As indicated by its name, pcPromoter-CNN is a CNN-based method for the identification and classification of sigma promoters and their sigma subclass and has been applied to *E. coli*. The results of the pcPromoter-CNN model were scrutinized by employed K-fold cross-validation technique as the value of K set by 5. Four performance evaluation metrics were used to record the remarkable outcomes of the model to compare with the state-of-the-art methods.

Several recent publications [20,22,23,24] have described standard rules for presenting promoter-related research results more effectively. We have used the five-step rules described by Chou’s [25] five-step rules, which are as follows:

- Selection and creation of benchmark dataset
- Numerical expression of dataset and DNA Sequence
- Proposal of powerful prediction architecture
- Performance evaluation of predictor using cross-validation
- Development of a web server to provide public access to predictor

**Figure 1:**
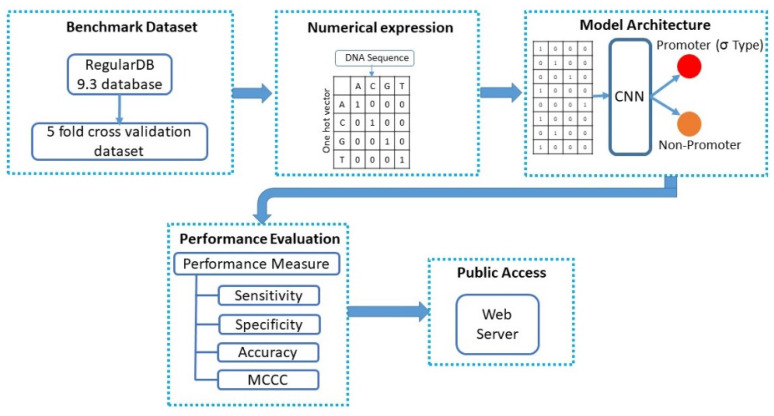
Model overview of pcPromoter-convolutional neural network (CNN).

A graphical representation of the five steps is presented in Figure 1. The remaining parts of this paper follow the research flow indicated by the steps presented by Chou’s rules.

## 2. Benchmark Dataset

To develop an efficient biological predictor, it is important to select a suitable benchmark dataset on which the proposed predictive model can be evaluated. The promoter sequence of *E. coli* used for the evaluation in this study is the same as that used in the studies of [19,20]. All of the promoter sequences have been sub-divided into sub-types. The length of each sequence in the dataset is 81 base pairs (bp). To utilize an improved quality dataset, this study has used the experimentally confirmed promoter sample data presented in version 9.3 of RegulonDB [26].

Furthermore, the non-promoter sequences have been collected from the middle of the long sequence of the *E. coli* K-12 genome. The dataset is redundant; thus, it can be biased toward one sigma class; therefore, CD-HIT software [11], in which the identity index was set at 0.8, was used to remove the redundancy. New samples of promoters were introduced in RegulonDB version 10.7 [27]. These samples were used as an independent test dataset. This independent dataset has promoter sequences only while no non-promoter sequences were introduced. Table 1 shows the further information regarding the number of sequences for each class in benchmark dataset as well as in the independent test dataset.

In the prepared benchmark dataset, there are two overall classes, and the whole dataset can be expressed as

$$D_{S}=\;P_{S}\;\cup \;N_{S}$$

where *D_S_*, is the overall benchmark dataset, *P_S_* represents the positive promoters and *N_S_* represent the negative promoters. The positive promoters are divided into six subclasses σ70, σ38, σ32, σ28, and σ24. Thus, *P_S_* can be further defined as

$$P_{S}=\sigma 70\cup \sigma 38\cup \sigma 32\cup \sigma 28\cup \sigma 24$$

## 3. Numerical Expression of DNA Sequence

A DNA sequence consists of four nucleotides (A, T, C and G). To perform numerical operations on the input DNA sequences, the sequences need to be converted to a numerical form. For this purpose, we have used one-hot encoding, where each nucleotide is converted to a four-element vector of which a single element is kept as 1 and all other elements are 0. The corresponding numerical representation to each nucleotide is

A (1, 0, 0, 0) T (0, 1, 0, 0) C (0, 0, 1, 0) G (0, 0, 0, 1)

After converting into this one-hot encoding numerical format, every DNA sequence was converted to an 81 × 4 two-dimensional matrix.

## 4. Proposed Methodology

### 4.1. Model Setup

The purpose of pcPromoter-CNN is to predict the presence of a promoter or a non-promoter within a queried DNA sequence, and if a promoter is identified, the next task is to identify the sigma class to which the promoter belongs. The dataset used to train the model is imbalanced, so different techniques, such as Synthetic Minority Oversampling Technique (SMOTE), were used to overcome the problem; however, SMOTE can easily turn the model toward data overfitting. Being inspired from [21] we proposed the use of a cascading binary classifier. The problem identified in the proposed architecture of [21] was that it uses four different encoding schemes and a large number of convolutional filters, which eventually increases both computational cost and complexity. In contrast, although the pcPromoter-CNN approach uses one encoding technique, a simple CNN architecture, and a small number of training parameters, it resulted in a performance improvement.

**Figure 2:**
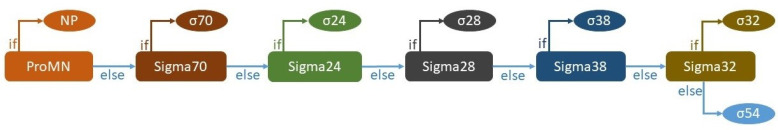
Model overview of pcPromoter-CNN.

The pcPromoter-CNN first identifies whether the input sequence is a promoter or non-promoter. If the input sequence is identified as a promoter, the next step is to identify its subclass. For subclass identification, we developed a mechanism where one after another subclass is selected for performing classification. For example, if σ70 is considered a positive class all other remaining subclasses are considered negative. If the test sequence is not classified as σ70, then the next subclass is selected as the positive class, σ70 is excluded from the list, and the other remaining subclasses are deemed negative. This process is carried out until the identification of the subclass of the promoter sequence is accomplished. Figure 2 and Table 2 presents detail on how this cascading process works.

### 4.2. Proposed CNN Architecture

The CNN is a computational model that uses different layers to learn a dataset’s features through various degrees of deliberation [28]. These models have accomplished outstanding results in different fields, generally because of the ongoing improvement of convolutional neural networks. CNNs achieved record-breaking results in medical image processing [29,30] and in computational biology [31,32,33,34,35,36,37]. Moreover, there are several remarkable examples of the use of CNNs to produce a prediction system that can identify the effects of genetic variation. The leading advantage of a CNN is that it does not require prior feature extraction; a CNN-based model can directly extract features from data. In this study, we have used this advantage of CNN to extract features directly from the base DNA sequence information.

**Figure 3:**
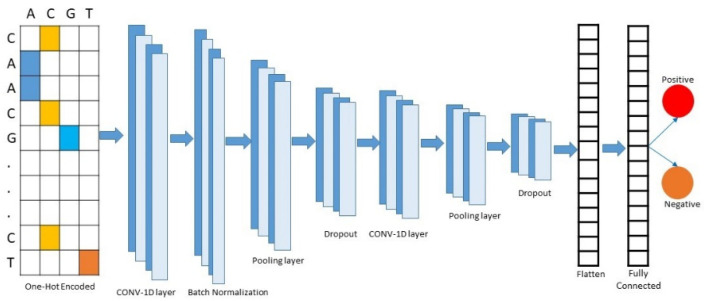
Model architecture.

Figure 3 shows the proposed CNN architecture for the classification of promoters and non-promoters. Through that architecture, the encoded sequence is passed to the input layer of the model. The model consists of two single-dimensional convolutional layers. The first convolution layer is followed by a batch normalization average-pooling and a dropout layer, while the second convolution layer is followed by average-pooling and a dropout layer.

The features extracted from the convolution layers are flattened using s flatten layer. After which the feature set proceeds to the fully connected dense layers for classification. For the selection of finest parameters for convolution, pooling, dropout, and dense layering, hyper-parameter tuning is carried out. Table 3 shows the range of hyper-parameters used for tuning purpose.

The first convolution layer uses 32 filters of kernel size 7, while the second convolution layer uses 32 filters with the smaller kernel size of 5. Batch normalization is performed on the first convolution layer. Moreover, both average-pooling layers use a pool size of 2 with 2 strides. The first dropout layer drops 35% of the features while the second dropout layer drops 30% of the features, thereby allowing the finest feature vector to be obtained. The convolution layers use a ReLU activation function that can be mathematically represented as

F(x)=max(0,x)

In the two fully connected layers, the first layer has 16 neurons and uses a ReLU activation function. In contrast, the second layer has a single neuron and a sigmoid activation function. The sigmoid function is represented as

$$S\left(x\right)=\frac{1}{1+e^{-x}}$$

All convolution layers and the dense layer use L2 regularization to control the overfitting problem. The loss function used in the model is a binary cross-entropy function. A stochastic gradient descent with 0.95 momentum and a learning rate of 0.007 is used as an optimizer. The mathematical expression for the binary cross-entropy function is

$$H_{p}\left(q\right)=-\frac{1}{(N_{pos}+N_{neg})\;}\lfloor \overset{N_{pos}}{\underset{i=1}{\sum }}\log\left(p\left(y_{i}\right)\right)+\overset{N_{neg}}{\underset{i=1}{\sum }}\log\left(1-p\left(y_{i}\right)\right)\rfloor$$

## 5. Results and Discussion

This section discusses the evaluation metrics and the performance achieved by the pcPromoter-CNN.

### 5.1. Evaluation Metrics

A five-fold cross-validation was utilized to examine the classification performance of the proposed model. To evaluate the performance of pcPromoter-CNN, we used four different metrics that have been previously used in other state-of-the-art techniques. These metrics are sensitivity (*Sn*), specificity (*Sp*), accuracy (*Acc*), and Matthews correlation coefficient (*MCC*). The mathematical expressions for these metrics are

$$MCC=\frac{TP\times TN-FP\times FN}{\sqrt{\left(TP+FP\right)\left(TP+FN\right)\left(TN+FP\right)\left(TN+FN\right)}}\;Acc=\frac{TP+TN}{TP+TN+FP+FN}\;Sp=\frac{TN}{TN+FP}\;Sn=\frac{TP}{TP+FN}$$

In the above equations, *TP*, *TN*, *FP* and *FN* represent the number of true positives, true negatives, false positives and false negatives, respectively.

### 5.2. Performance Evaluation

To evaluate the performance of the proposed model we carried out k-fold validation in which the value of k was 5. The tests were similarly carried out using IPromoter2L, IPromoter-BnCNN, and MULTiPly, which are considered state-of-the-art techniques to diagnose and classify *E. coli* sigma promoters. Table 4 shows the pcPromoter-CNN results for the classification between promoter and non-promoter and a comparison with the results of the state-of-the-art techniques. The pcPromoter-CNN achieved 89.84% sensitivity, 90.38% specificity, 90.11% accuracy and 0.802 MCC. The pcPromoter-CNN exhibited improved performance in all four parameters when compared with IPromoter2L, IPromoter-BnCNN and MULTiPly. A significant improvement of 3.9% for the value of MCC shows how accurately the proposed technique distinguishes the promoter and non-promoter class.

A summary of the performance evaluation results for the next step in pcPromoter-CNN is presented in Table 5. The table summarizes the proposed method’s identification of each of the five promoter sigma subclasses. The proposed model has depicted an increase in overall accuracy by 3%, 2.8%, 7.3%, 4.9% and 4.8% for σ24, σ28, σ32, σ38, and σ70 respectively. For all subclasses, the pcPromoter-CNN achieved notable increases in terms of specificity.

We used independent test dataset to further evaluate the performance of pcPromoter-CNN. Table 6 shows the comparison results of pcPromoter-CNN with state-of-the-art methods. Independent test dataset doesn’t contain non-promoter sequences that is why we only reported the values of true positive and false negative. Except for the promoter σ24, pcPromoter-CNN shows promising results compared to state-of-the-art methods.

**Figure 4:**
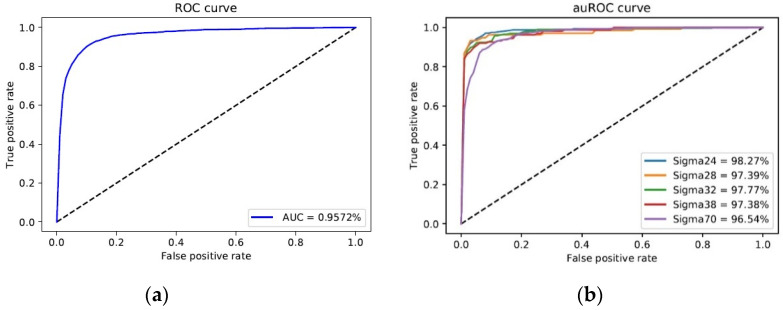
Receiver operating characteristic (ROC) curves. (**a**) Promoter non-promoter ROC curve. (**b**) Sigma subclasses ROC curve.

Figure 4a illustrates the receiver operating characteristic (ROC) curve for the promoter and non-promoter predication and Figure 4b presents an ROC curve for the promoter subclasses classification; the curve clearly shows a large area under the curve (AUC; 95.7%). The five promoter subclasses assessed showed similarly high AUC values (96.5–98.3%).

## 6. Webserver

**Figure 5:**
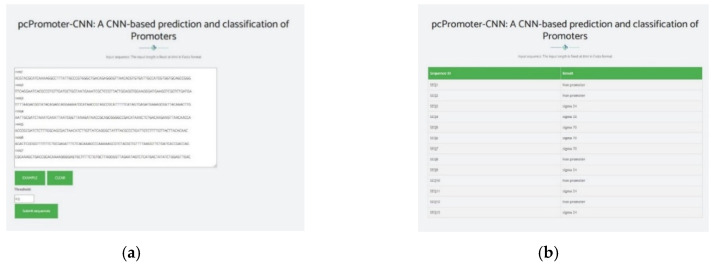
Webserver. (**a**) Insertion of sequences for prediction. (**b**) Predictor output.

To provide easy access to the proposed tool for the research community, a web server that hosts the high performing pcPromoter-CNN tool is freely available at http://nsclbio.jbnu.ac.kr/tools/pcPromoter-CNN/. Many researchers [38,39,40] have followed this step. The pcPromoter-CNN is a user-friendly tool that can be used by researchers and experts in the fields of medicine and bioinformatics. It supports two options which are, direct sequence input and uploading a file containing sequences for prediction. The length of each sequence should be 81 nt containing A, C, G, and T. In the case of uploading a file, the maximum number of sequences for prediction is 1000. Figure 5 shows a snippet from the web-server where Figure 5a shows an example of inserting sequences for prediction and Figure 5b shows the output of the predictor. Furthermore, the code of pcPromoter-CNN was made available at https://github.com/Shujaatmalik/pcPromoter-CNN.

## 7. Conclusions

The classification of the promoter and non-promoter DNA sequences is an important task in the fields of medicine and bioinformatics. Furthermore, knowledge of the sigma subclass classification of a promoter DNA sequence can have a significant role in elucidating various biological aspects of a promoter. To assist in such endeavors, we propose the use of the pcPromoter-CNN tool. The tool is capable of efficiently classifying a DNA sequence as a promoter or non-promoter and can identify the sigma subclass of a promoter. The CNN-based tool uses a single encoding scheme for classification, and its proposed architecture was evaluated by using a publicly available dataset. Overall, the tool produced notable classification improvements over the results obtained using existing techniques.

## Figures and Tables

**Table 1:** Sigma classes and sample size.

Classes	Benchmark Dataset	Independent Test Dataset
Promotor	2860	256
Non-Promotor	2860	0
σ70	1694	199
σ54	94	0
σ38	163	10
σ32	291	13
σ28	134	04
σ24	484	30

**Table 2:** Cascade binary classifiers.

Binary Classifier	Positive Class	Negative Class
ProMN	Promoter	Non-Promoter
Sigma70	σ70	σ54, σ38, σ32, σ28, σ24
Sigma24	σ24	σ54, σ38, σ32, σ28,
Sigma28	σ28	σ54, σ38, σ32,
Sigma38	σ38	σ54, σ38
Sigma32	σ32	σ54

**Table 3:** Hyper-parameter tuning parameters.

Number Convolution Filters	8, 16, 32, 64, 128
Convolution Kernel Size	3, 5, 7, 9, 11
Pooling Layer Kernel Size	2, 4
Dropout Ratio	0.15, 0.20, 0.25, 0.30, 0.35, 0.40, 0.45
Dense Layer Neurons	8, 16, 32, 64

**Table 4:** Promoter and non-promoter identification comparison using five-fold cross-validation on benchmark dataset.

Methods	Sn (%)	Sp (%)	Acc (%)	MCC
IPromoter-2L	79.2	84.2	81.7	0.637
MULTiPly	87.3	86.6	86.9	0.739
IPromoter-BnCNN	88.3	88.0	88.2	0.763
pcPromoter-CNN	89.84	90.38	90.11	0.802

**Table 5:** Sigma promoter performance comparison. ‘Pc’ represents the results of proposed architecture, ‘Bn’ represents the results of iPromoter-BnCNN, ‘Mu’ represents results of MULTiPly architecture.

Metrics	σ24			σ28			σ32			σ38			σ70		
	Pc	Bn	Mu	Pc	Bn	Mu	Pc	Bn	Mu	Pc	Bn	Mu	Pc	Bn	Mu
Acc (%)	96.8	93.8	91.2	98.9	96.1	95.9	97.9	90.6	85.7	96.5	91.6	85.3	92.1	87.3	84.9
Sn (%)	88.5	93.3	88.8	84.6	97.8	95.9	87.7	91.7	82.2	87.2	94.9	83.3	94.9	91.0	90.4
Sp (%)	98.5	94.1	92.9	99.6	93.6	91.3	99.0	89.8	88.4	98.9	89.3	86.7	87.9	82.2	76.9
MCC	0.885	0.873	0.818	0.875	0.918	0.876	0.881	0.90	0.708	0.882	0.833	0.699	0.836	0.737	0.668

**Table 6:** Validation of pcPromoter-CNN on independent test dataset. ’TP’ represents true positives and ’FN’ represents false negatives.

Parameter	Promoter			σ24			σ28			σ32			σ38			σ70		
	Pc	Bn	Mu	Pc	Bn	Mu	Pc	Bn	Mu	Pc	Bn	Mu	Pc	Bn	Mu	Pc	Bn	Mu
TP	236	245	238	24	28	19	2	1	0	12	10	5	6	3	4	180	179	180
FN	20	11	18	6	2	11	2	3	4	1	3	8	4	7	6	19	20	19
